# E47 upregulates ΔNp63α to promote growth of squamous cell carcinoma

**DOI:** 10.1038/s41419-021-03662-3

**Published:** 2021-04-08

**Authors:** Jing Xu, Fengtian Li, Ya Gao, Rongtian Guo, Liangping Ding, Mengyuan Fu, Yong Yi, Hu Chen, Zhi-Xiong Jim Xiao, Mengmeng Niu

**Affiliations:** Center of Growth, Metabolism and Aging, Key Laboratory of Bio-Resource and Eco-Environment, Ministry of Education, College of Life Sciences, Sichuan University, Chengdu, China

**Keywords:** Cell growth, Transcription

## Abstract

Targeted therapy has greatly improved both survival and prognosis of cancer patients. However, while therapeutic treatment of adenocarcinoma has been advanced greatly, progress in treatment of squamous cell carcinoma (SCC) has been slow and ineffective. Therefore, it is of great importance to decipher mechanisms and identify new drug targets involved in squamous cell carcinoma development. In this study, we demonstrate that E47 plays the distinctive and opposite roles on cell proliferation in adenocarcinoma and squamous cell carcinoma. While E47 suppresses cell proliferation in adenocarcinoma cells, it functions as a oncoprotein to promote cell proliferation and tumor growth of squamous cell carcinoma. Mechanistically, we show that E47 can directly bind to the promoter and transactivate ΔNp63 gene expression in squamous cell carcinoma cells, resulting in upregulation of cyclins D1/E1 and downregulation of p21, and thereby promoting cell proliferation and tumor growth. We further show that expression of E2A (E12/E47) is positively correlated with p63 and that high expression of E2A is associated with poor outcomes in clinical samples of squamous cell carcinoma. These results highlight that the E47-ΔNp63α axis may be potential therapeutic targets for treatment of squamous cell carcinoma.

## Introduction

Lung cancer is the leading cause of cancer-related death worldwide. Non-small cell lung cancer (NSCLC) accounts for 85% of all cases of lung cancers^1,2^. NSCLC is mainly comprised of adenocarcinoma and squamous cell carcinoma (SCC), both of which exhibit unique characters including tumor origins, causation, and genomic alterations^3–5^. Activating mutations in epidermal growth factor receptor (EGFR) and anaplastic lymphoma kinase (ALK) rearrangement are the major driving force for lung adenocarcinoma while most squamous cell carcinomas display somatic mutation of p53^4,6–8^. In recent years, EGFR-targeted and ALK-targeted therapies have been developed and have greatly improved clinical outcomes of patients with lung adenocarcinoma^9–11^. However, there are lack of specific targeted therapies for treatment of squamous cell carcinoma. Patients with squamous cell carcinoma, including lung squamous cell carcinoma (LUSC) and head and neck squamous cell carcinoma (HNSCC), suffer significantly poorer overall survival than those with adenocarcinoma^12–19^. Therefore, it is of great importance to decipher unique mechanisms and identify drug targets involved in the development of squamous cell carcinoma.

It has been documented that p63, a member of p53 family, is frequently overexpressed in squamous cell carcinoma^4^. p63 consists of TAp63 and ΔNp63 isoforms due to two alternative transcription start sites at the N terminus. Furthermore, alternative splicing at the C terminus gives rise to five different C terminal isoforms (α, β, γ, δ, and ε) of p63^20–23^. Among these p63 protein isoforms, ΔNp63α is the major p63 protein isoform specifically expressed in epithelial cells. ΔNp63α can function as a proto-oncogene in promoting cancer cell proliferation and survival^24–27^. It has been well documented that ΔNp63α can directly bind to the promoter of CDKNIA (also known as p21CIP1) to inhibit its transcription, thereby promoting cell cycle progression and suppression of cell apoptosis^28–30^. ΔNp63α can also promote tumor growth by downregulation expression of growth-suppressing genes including PUMA and MIC-1^31,32^, and upregulation expression of growth-promoting genes including cyclins D1 and E1 (CCND1, CCNE1)^33,34^. In addition, p63 can form hetero oligomers with p53 and inhibits its transcription function^35^.

The TCF3 gene, also called E2A, encodes two alternative splicing variants, E47 and E12, both of which are members of basic helix–loop–helix (bHLH) transcription factor^36^. E47 can form homodimer or heterodimer to bind to the canonical E-Box response element (CANNTG) and regulate transcription of the target genes^37^. It has been shown that E47 plays a critical role in promoting B-cell lymphopoiesis, T-cell development, myogenesis, and cell proliferation^38–40^. On the other hand, it has been reported that E47 can function as a growth suppresser in adenocarcinoma^41,42^. Under that circumstances, E47 can downregulate Myc protein expression and upregulate expression of p27 or p21 protein to inhibit cell-cycle progression and promote senescence^43,44^. However, the role of E47 in squamous cell carcinoma is largely unclear. In this study, we demonstrate that E47 plays the inhibitory role on cell proliferation in adenocarcinoma. By sharp contrast, E47 functions as a oncoprotein to promote growth of squamous cell carcinoma through directly transactivating ΔNp63α expression.

## Results

### E47 plays distinctive and opposite roles in regulation cell proliferation of adenocarcinoma and squamous cell carcinoma

**Fig. 1:**
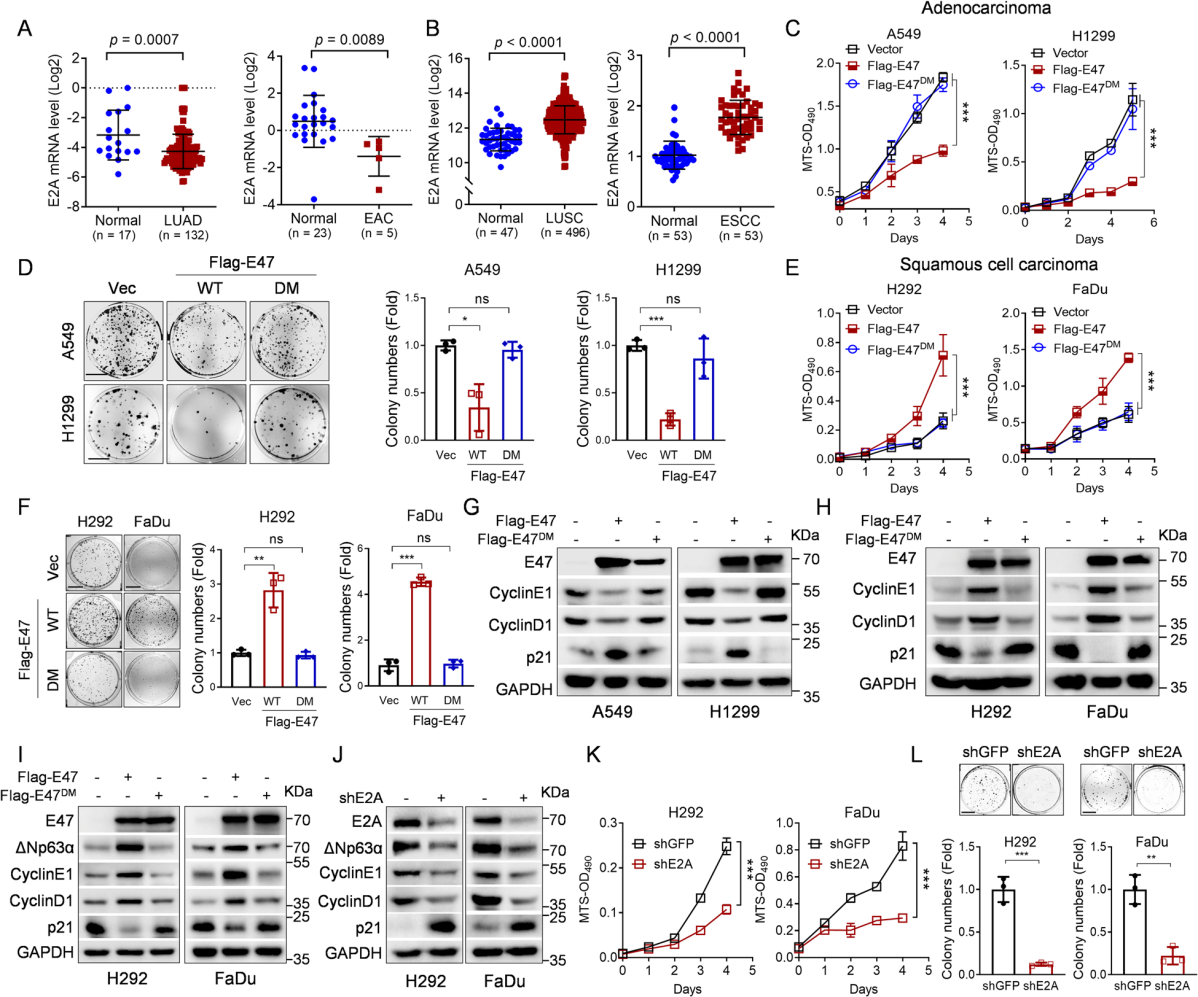
E47 upregulates ΔNp63α expression and promotes squamous carcinoma cell proliferation. **A** The Oncomine dataset “Hao_Esophagus” or “Bhattacharjee_Lung” was used to analyze E2A mRNA levels in normal tissues and human esophagus adenocarcinoma (EAC) or lung adenocarcinoma (LUAD). **B** The Oncomine dataset “Su_Esophagus” or “TCGA” was used to analyze E2A mRNA levels in normal tissues and human esophagus squamous cell carcinoma (ESCC) or lung squamous cell carcinoma (LUSC). **C**–**H** A549, H1299, H292, or FaDu cells stably expressing wild-type Flag-E47, Flag-E47^DM^ mutant (E47^A592N/I596D^) or a vector control were subjected to MTS assays (**C**, **E**), Colony formation analyses (**D**, **F**), or Western blot analyses (**G**–**H**). Scale bar = 1 cm. Three independent experiments were performed. Data were presented as mean ± SD. *p < 0.05, **p < 0.01, ***p < 0.001. **I** H292 or FaDu cells stably expressing wild-type Flag-E47, Flag-E47^DM^ mutant, or a vector control were subjected to Western blot analyses. **J**–**L** H292 or FaDu cells stably expressing shRNAs against E2A (+) or GFP (−) were subjected to Western blot analyses (**J**), MTS assays (**K**), or Colony formation analyses (**L**). Scale bar = 1 cm. Three independent experiments were performed. Data were presented as mean ± SD. **p < 0.01, ***p < 0.001.

It has been reported that E47 inhibits cell proliferation in adenocarcinoma, while its roles in SCC are largely unknown. In this study, we aimed to examine the roles of E47 on cell proliferation and tumor growth of SCC. With this regard, we first examined E47 protein expression in cancers utilizing Oncomine database. As shown in Fig. 1A, B, E47 was lowly expressed in adenocarcinoma of lung and esophagus, whereas E47 was highly expressed in SCC of lung and esophagus. Consistently, the expression of E47 in adenocarcinoma A549 cells was lower than that in SCC FaDu and H292 cells (Fig. S1A). Moreover, ectopic expression of E47 inhibited cell proliferation in adenocarcinoma A549 and H1299 cells, as evidenced by MTS assays and colony formation assays (Fig. 1C, D), while ectopic expression of E47 promoted cell proliferation in SCC H292, FaDu, and KYSE150 cells (Fig. 1E, F and Fig. S1B). Unlike wild-type E47, E47^A592N/I596D^ mutant (E47^DM^) defective in its transcriptional activity^45^, had little effects on cell proliferation (Fig. 1C–F and Fig. S1B). These data indicate that E47 plays the opposite roles on cell proliferation in adenocarcinoma and SCC and that E47 functions as a oncoprotein in SCC, which is dependent on its transcriptional activity. In keeping with this notion, E47 inhibited expression of Cyclin E1 and Cyclin D1 and upregulated expression of cell-cycle inhibitor p21 in adenocarcinoma A549 and H1299 cells (Fig. 1G). By sharp contrast, E47 significantly upregulated expression of Cyclin E1 and Cyclin D1 and suppressed expression of p21 in SCC H292 and FaDu cells (Fig. 1H).

We then investigated the molecular basis by which E47 promotes cell proliferation in SCC. It has been documented that overexpression of p63 is a typical feature of SCC^4^. We therefore asked whether ΔNp63α plays a role in E47-mediated stimulation of SCC cell proliferation. As shown in Fig. 1I and Fig. S1C, ectopic expression of E47 significantly upregulated ΔNp63α expression, concomitant with increased Cyclins D1/E1 expression and decreased p21 expression in H292, FaDu, and KYSE150 cells, while ectopic expression of E47^DM^ mutant was unable to do so. We then used shRNAs specific for E2A (E12/E47) to knockdown of E47 in H292, FaDu, and KYSE150 cells. As shown in Fig. 1J–L and Fig. S1D, E, silencing of E47 significantly reduced expression of ΔNp63α, concomitant with decreased Cyclins E1/D1 expression, increased p21 expression, and suppression of cell proliferation. Together, these results indicate that E47 significantly upregulates ΔNp63α protein expression and promotes cell proliferation in SCC cells.

### E47 promotes SCC cell proliferation and tumor growth via upregulation of ΔNp63α expression

**Fig. 2:**
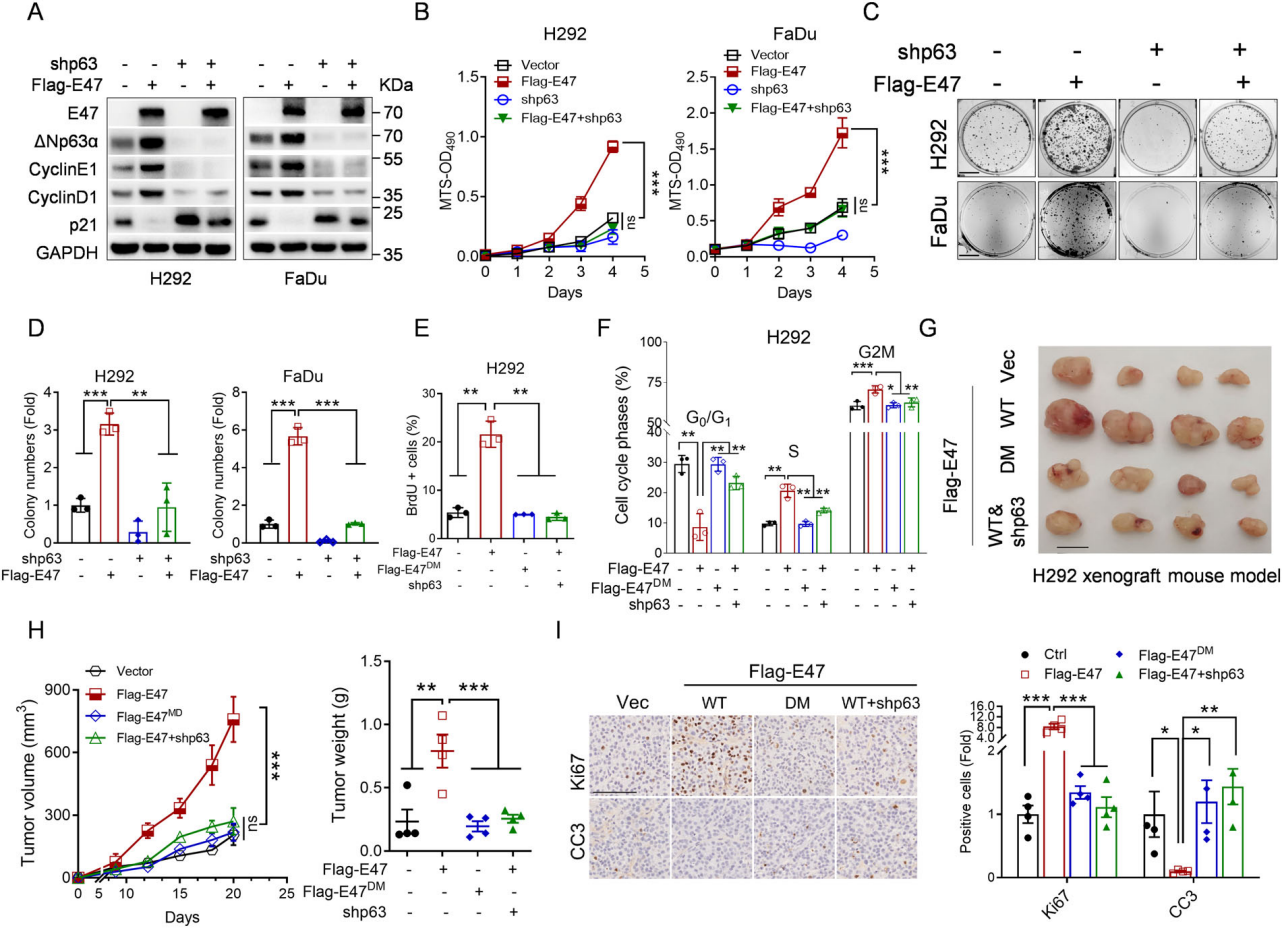
Knockdown of ΔNp63α blocks E47-mediated cell proliferation and tumor growth in vitro and in vivo. **A**–**D** H292 or FaDu cells stably expressing either Flag-E47 or shp63, or both were subjected to Western blot analyses (**A**), MTS assays (**B**), or Colony formation analyses (**C**, **D**). Scale bar = 1 cm. Three independent experiments were performed. Data were presented as mean ± SD. **p < 0.01, ***p < 0.001. **E**, **F** H292 cells stably expressing either Flag-E47 (wild-type or DM mutant) or shp63, or both were subjected to BrdU or PI staining and FACS analyses. Three independent experiments were performed. Data were presented as mean ± SD. *p < 0.05, **p < 0.01, ***p < 0.001. **G**, **H** H292 cells (1 × 10^6^) stable cells were subcutaneously injected into BALB/C nude mice (*n* = 4/group). Tumor sizes were measured every 2–3 days. Mice were euthanized by day 20 after inoculation and individual tumor weight was measured. Tumor photos, tumor volume, and tumor weight were presented. Scale bar = 1 cm. Data were presented as means ± SEM. **p < 0.01, ***p < 0.001. **I** Tumors were fixed, embedded in paraffin, sectioned, and IHC was performed for Ki67 and cleaved caspase-3 (CC3). Scale bar = 100 μm. Data were presented as means ± SEM. *p < 0.05, **p < 0.01, ***p < 0.001.

To investigate the causative role of ΔNp63α in E47-induced cell proliferation, we performed the rescuing experiments. As shown in Fig. 2A, silencing of ΔNp63α completely reversed E47-induced changes expression of Cyclin E1, Cyclin D1, and p21, all of which are downstream effectors of ΔNp63α. Consistently, silencing of ΔNp63α significantly rescued E47-mediated cell proliferation in H292 and FaDu cells, as evidenced by MTS assays and colony formation assays (Fig. 2B–D). In addition, ectopic expression of wild-type E47, but not E47^DM^, led to increased BrdU+ cell and S-phase population in SCC H292 cells, both of which were rescued by simultaneous silencing of ∆Np63 (Fig. 2E, F and Fig. S2A). By contrast, silencing of E47 led to an increase of the BrdU+ cells in H1299 cells (Fig. S2B). These results indicate that ΔNp63α plays a causative role in E47-induced cell proliferation of SCC.

To further substantiate this conclusion, we examined the role of E47-ΔNp63α in tumor growth in vivo. As shown in Fig. 2G, H, ectopic expression of E47, but not E47^DM^, dramatically promoted tumor growth in H292 xenograft mouse model. Notably, silencing of ΔNp63α completely rescued E47-induced tumor growth (Fig. 2G, H). Furthermore, Flag-E47-expressing tumors exhibited significant increased Ki67+ cells and decreased cleaved caspase-3 + (CC3 + ) cells, all of which were rescued by silencing of ΔNp63α (Fig. 2I), indicating that E47-mediated upregulation of ΔNp63α consequently facilitates tumor growth through increased cell proliferation and reduced apoptotic cell death. Together, these results demonstrate that ΔNp63α is an essential downstream effector of E47 in promoting cell proliferation and tumor growth in SCC.

### ΔNp63 is a direct E47 transcriptional target

**Fig. 3:**
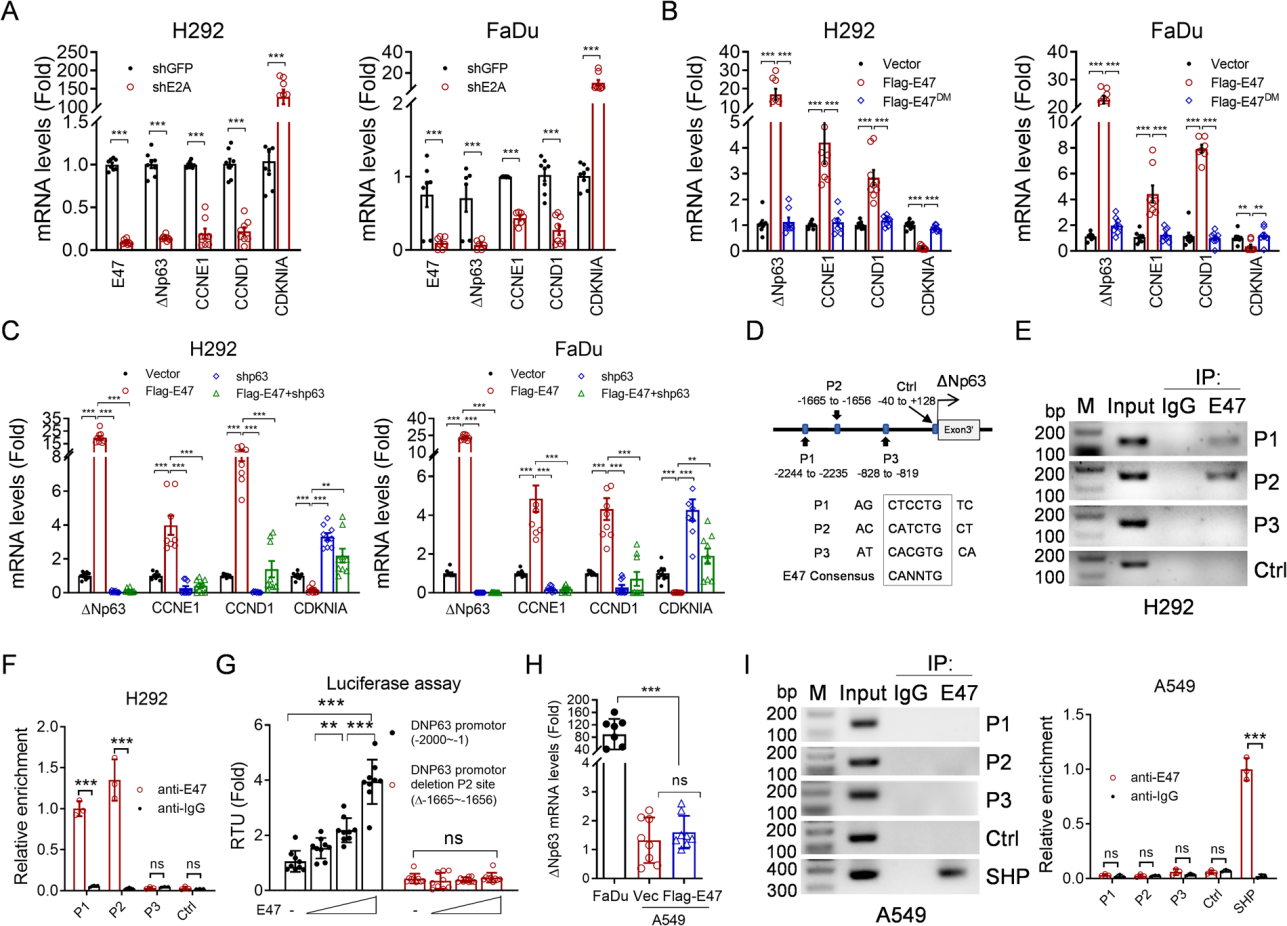
Wild-type E47, but not E47^DM^ mutant, directly transactivate ΔNp63α. **A** H292 or FaDu cells stably expressing shRNAs against E2A or control were subjected to Q-PCR analyses. Three independent experiments were performed. Data were presented as mean ± SD. ***p < 0.001. **B** H292 or FaDu cells stably expressing wild-type E47, E47^DM^ mutant, or a vector control were subjected to Q-PCR analyses. Three independent experiments were performed. Data were presented as mean ± SD. **p < 0.01, ***p < 0.001. **C** H292 or FaDu cells stably expressing either Flag-E47 or shp63, or both were subjected to Q-PCR analyses. Three independent experiments were performed. Data were presented as mean ± SD. **p < 0.01, ***p < 0.001. **D** Three putative E47-binding elements (P1: − 2244 to −2235; P2: −1665 to −1656; P3: −828 to −819) and a negative control (NC: −40 to +128) on *ΔNTp63* gene promoter were predicted by JASPAR software and E47-binding consensus sequence are depicted. **E**–**F** ChIP assays were performed in H292 cells using a specific E2A antibody (anti-E47) or a control rabbit normal IgG (anti-IgG) and primers specific for P1, P2, and P3. A randomly segment (−40 to +128) on ΔNp63 gene promoter was used as negative control (Ctrl). Q-PCR analyses of ChIP samples were performed. ***p < 0.001. **G** HEK293T cells were co-transfected with 0 ng, 100 ng, 200 ng, or 400 ng E47 and 100 ng ∆Np63-Gluc-SEAP reporter or its deletion mutation (∆ −1665~−1656) expressing plasmid for 36 h, and then the cell culture media were collected and ∆Np63-Gluc and SEAP activities were measured. The ∆Np63-Gluc activity was normalized to SEAP activity. Three independent experiments were performed. Data were presented as mean ± SD. **p < 0.01, ***p < 0.001. **H** FaDu or A549 cells stably expressing wild-type E47 or a vector control were subjected to Q-PCR analyses. Three independent experiments were performed. Data were presented as mean ± SD. ***p < 0.001. **I** ChIP assays were performed in A549 cells using a specific E2A antibody (anti-E47) or a control rabbit normal IgG (anti-IgG) and primers specific for P1, P2, and P3. A randomly segment (−40 to +128) on ΔNp63 gene promoter was used as negative control (Ctrl). SHP (−474 to −120) was used as a positive control. Q-PCR analyses of ChIP samples were performed. ***p < 0.001.

Since our aforementioned results show that E47-mediated upregulation of ΔNp63α expression is dependent on its transcriptional activity, we then investigated whether *E4*7 can regulate ΔNp63α gene transcription. As shown in Fig. 3A, silencing of E2A significantly reduced steady-state ΔNp63 mRNA levels, concomitant with upregulation of steady-state p21 (*CDKN1A*) mRNA levels and downregulation of steady-state mRNA levels of Cyclin E1 (*CCNE1*) and Cyclin D1 (*CCND1*). Conversely, ectopic expression of E47 dramatically upregulated steady-state ΔNp63 mRNA levels, concomitant with the changes of steady-state mRNA levels of Cyclin E1, Cyclin D1, and p21 in H292 and FaDu cells (Fig. 3B). Unlike wild-type E47, E47^DM^ mutant failed to do that (Fig. 3B). Furthermore, silencing of ΔNp63α significantly rescued E47-mediated changes of steady-state mRNA levels of Cyclin E1, Cyclin D1 and p21 in H292 and FaDu cells (Fig. 3C). Together, these results indicate that E47 upregulates ΔNp63 gene transcription, which, in turn, regulates transcription of cyclins D1/E1 and p21.

To examine whether ΔNp63α is a direct E47 transcriptional target, we performed ChIP assay. As shown in Fig. 3D, the ΔNp63 promoter sequence was analyzed by JASPER (http://jaspar.genereg.net), which revealed three putative E47 binding sites, including P1 (−2244 to −2235); P2 (−1665 to −1656) and P3 (−828 to −819) that match to the consensus sequence of E47 binding sites CANNTG^37^. Data from ChIP experiments showed that E47 directly bound P1 and P2 but not P3 sites in H292 cells (Fig. 3E, F). In addition, luciferase reporter assays showed that ectopic expression of E47 significantly enhanced ΔNp63-Gluc reporter (−2000~−1) activities in a dose-dependent manner, but failed to enhance the activities of ΔNp63-Gluc reporter with a deletion of P2 site (∆ −1665 to −1656) (Fig. 3G). These results demonstrate that E47 can directly bind to the promoter and transactivate ΔNp63 gene expression in squamous cell carcinoma cells.

We further examined whether E47 could bind to p63 promoter and transactivate p63 gene expression in adenocarcinoma cells. As shown in Fig. 3H, ectopic expression of E47 was unable to upregulate steady-state ΔNp63 mRNA levels in A549 cells. In addition, while E47 bound small heterodimer partner (SHP), a known target of E47^46^, as expected, it failed to bind to any of three putative E47 binding sites on ΔNp63 promoter in A549 cells (Fig. 3I). These results indicate that ΔNp63α is a direct E47 transcriptional target in SCC cells, but not in adenocarcinoma cells.

### E2A is positively correlated with p63 expression in SCC and its high expression is associated with poor overall survival of SCC patients

**Fig. 4:**
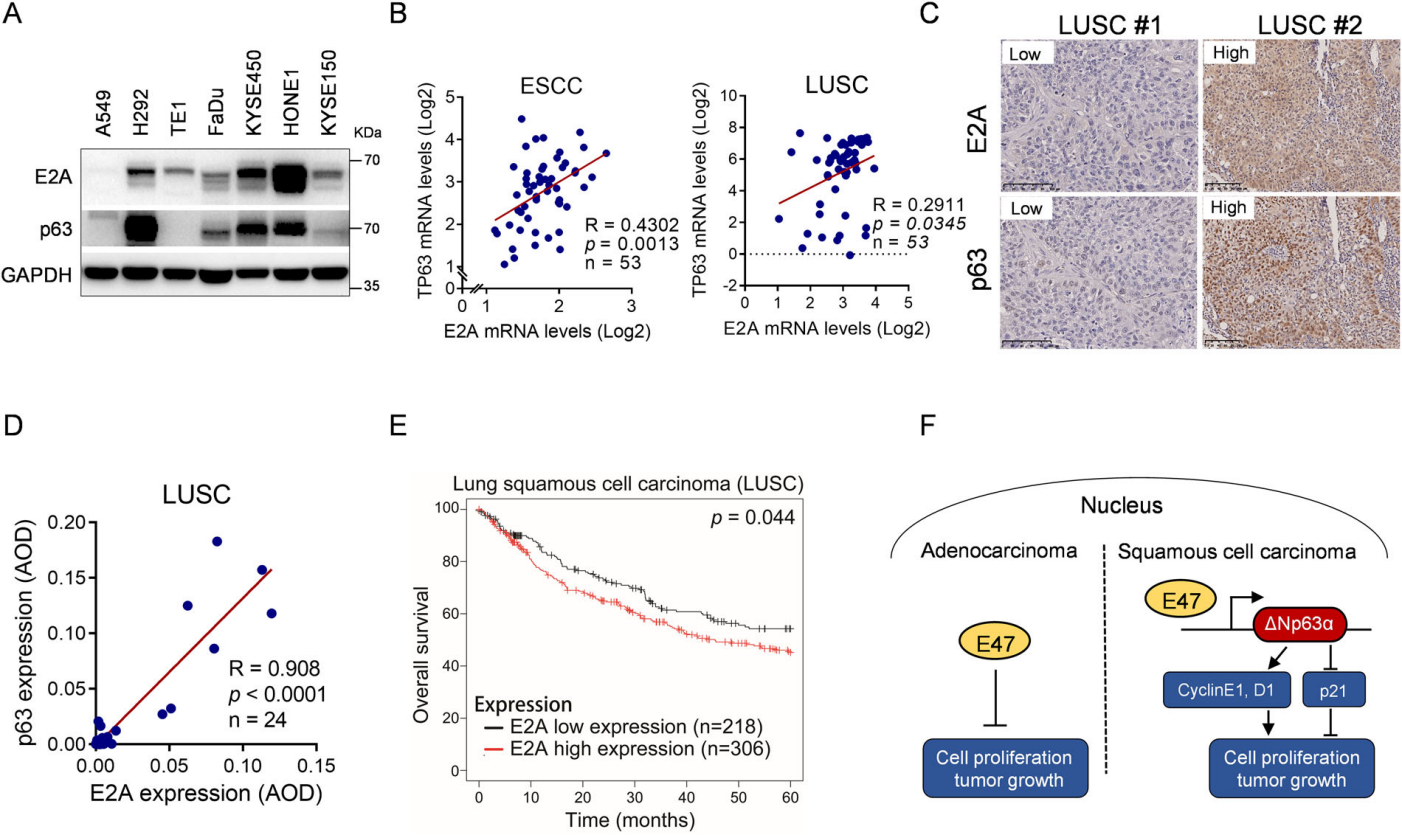
E47 positively correlates with ΔNp63α expression and its high expression is associated with poor overall survival in human lung squamous cell carcinoma. **A** Adenocarcinoma A549 and SCC H292, TE1, FaDu, KYSE450, KYSE150, or HONE1 cells were subjected to Western blot analyses. **B** The Oncomine datasets “Su_Esophagus” and “Bild_Lung” were used to analyze Pearson correlation of E47 (E2A) and p63 expression in ESCC and LUSC. **C**–**D** Consecutive tissue microarray slides derived from human lung squamous cell carcinoma (HLug-S030PG02) were subjected to IHC analysis for Pearson correlation of E47 (E2A) and p63. p63 antibody used here can recognize all isoforms of p63 (CY5659, Abways, Shanghai, China). Representative images were shown. Data were quantified by average optical density (AOD). Scale bar = 100 μm. **E** Kaplan–Meier plots of overall survival of human squamous cell carcinoma patients were stratified by the E2A mRNA expression levels in the patient tumor samples. **F** A working model depicting that the opposite roles of E47 on cell proliferation in adenocarcinoma and squamous cell carcinoma. E47 can function as a pro-oncogene by targeting ΔNp63 promoter to promote it transcription, resulting in promoting cell proliferation and tumor growth in lung squamous cell carcinoma.

Our results prompted us to verify the clinical relevance of the E47-p63 axis in squamous cell carcinoma. As shown in Fig. 4A, a positive correlation between E47 and ∆Np63 protein levels was observed in 4 out of 6 SCC cell lines examined. In addition, oncomine analyses showed that E2A (E12/E47) mRNA levels were positively correlated with p63 mRNA levels in esophagus squamous cell carcinoma (ESCC) (*R* = 0.4302; *p* = 0.0013) and in lung squamous cell carcinoma (LUSC) (*R* = 0.2911; *p* = 0.0345) (Fig. 4B). To further investigated the clinical relevance of the E47-p63 axis, we performed immunohistochemical (IHC) analyses of human LUSC tissue microarrays (*n* = 24). As shown in Fig. 4C, D, a significant positive correlation was observed between p63 and E2A protein expression (*R* = 0.908; *p* < 0.0001). In addition, clinical analysis of Kaplan–Meier dataset showed that LUSC patients with high expression of E2A exhibited poor overall survival (OS) (Fig. 4E). Together, these results indicate that E47 is positively correlated with p63 in SCC and that increased E47 expression is associated with poor clinical outcomes of SCC patients.

## Discussion

In recent years, targeted therapy to treat adenocarcinoma has greatly improved patient outcomes. However, little progress has been made on targeted therapy for squamous cell carcinoma (SCC). It is urgently needed to decipher mechanisms and identify drug targets involved in the development of SCC.

There are evidently unique genomic alterations in adenocarcinoma (ADC) and SCC. One of the most differentially expressed genes between ADC and SCC is p63^47^. p63 is often overexpressed in SCC and is a well-known marker of squamous differentiation, whereas p63 is little expressed in advanced adenocarcinoma. In this study, we demonstrate the opposite roles of E47 on regulation of growth of ADC and SCC, in which ΔNp63α serves as a key mediator. Most significantly, E47 can directly bind to the ΔNp63α promoter in SCC H292 cells, but not in ADC A549 cells, consistent with the observations that A549 cells express very little ΔNp63α and that E47 is unable to transactivate ΔNp63α in A549 cells. The molecular mechanisms are not clear, but it is plausible that there may be lack of transcription-cofactor(s) required for E47 DNA-binding to the ΔNp63α promoter in A549 cells or that the epigenic landscape at the chromatin state (histone acetylation/methylation) at ∆Np63 promoter is fundamentally different in ADC and SCC. Notably, our study is in line with the observations that the increased p63 expression promotes progressive transdifferentiation of lung ADC to SCC in mouse models^48,49^. Therefore, we propose that the expression of ΔNp63α is a critical intrinsic link between ADC and SCC. It would be worthwhile to further investigate how the expression of ΔNp63α is lost during the development of ADC or expression of ΔNp63α is established during SCC development and whether E47 plays a role in transdifferentiation of lung ADC to SCC.

p63 plays an important role in regulation of a variety of biological processes, including embryonic development, differentiation, tumor growth, aging, tumor metastasis, and tumorigenesis^23^. It has been shown that p63 gene expression can be upregulated by several transcriptional factors. Upon keratinocyte differentiation, transcriptional factors C/EBPα and DEC1 can target ΔNp63 promoter to increase ΔNp63α transcription^50^. Oct4 can target p63 promoter and stimulates p63 to promote cell transformation^51^. In addition, WT1 can bind to the p63 promoters and activate its expression to promote cell proliferation^52^. We have recently shown that FOXO3a can also directly transactivate ΔNp63α during cancer metastasis^25^. In this study, we have identified that E47 is a novel direct transcription factor of ΔNp63. E47 can bind to ΔNp63 promoter and increase its expression, thereby regulating expression of ΔNp63α targeted genes involved in cell proliferation, including cyclins D1/E1 and p21. We demonstrate that ΔNp63α protein is a critical target of E47 in promoting cell proliferation and tumor growth in squamous cell carcinoma.

Importantly, clinical validation of human lung SCC and esophagus SCC samples reveals a significant and positive correlation between p63 and E47, and that lung SCC patients with high E47 expression show poor overall survival. Therefore, targeting E47-ΔNp63α axis may be a potential therapeutic strategy for treatment of squamous cell carcinoma.

## Materials and methods

### Cell culture

Human lung cancer cells H292, esophageal squamous cell carcinoma cells KYSE150, head and neck squamous carcinoma cells FaDu, lung adenocarcinoma cells H1299/A549, and human embryonic kidney cells HEK293T were obtained from the American Type Culture Collection (ATCC). H292 and KYSE150 cells were grown in RPMI-1640 medium (Invitrogen, Carlsbad, CA, USA), supplemented with 10% FBS (Hyclone, Logan, UT, USA). FaDu, H1299, A549, and HEK293T cells were grown in DMEM medium (Invitrogen, Carlsbad, CA, USA), supplemented with 10% FBS (Hyclone, Logan, UT, USA). All cells were grown in the medium containing 1% penicillin G/streptomycin sulfate (GIBCO, Rockville, MD, USA) and were incubated at 37 °C in a humidified incubator under 5% CO_2_. Authentication of cells was verified by short tandem repeat DNA profiling.

### Plasmids and lentiviral infection

Human E47 cDNA was cloned into the plvx-puro lentiviral vector. Human ∆Np63 promotor (−2000~−1) was cloned into the GLuc-ON promoter reporter vector. E47^A592N/I596D^ and ∆Np63 promotor deletion P2 site (Δ−1665~−1656) were generated by KOD-Plus-Mutagenesis kit (SMK-101, Toyobo Osaka). A pLKO.1-puromysci lentiviral vector was used to generate short hairpin RNAs (shRNAs) targeting GFP, E2A, or p63. Specific oligos of shRNA are used as follows. shGFP: 5′-GAAGCAGCACGACTTCTTC-3′; shE2A: 5′-AGCAGCCTCTCTTCATCC-3′; shp63: 5′-GAGTGGAATGACTTCAACTTT-3′. All the constructs were confirmed by direct DNA sequencing. Recombinant lentiviruses were amplified in HEK293T cells as described^53^.

### Western blotting analysis

Cells were lysed in the EBC250 lysis buffer (250 mM NaCl, 25 mM Tris pH 8.0, 0.5% Nonidet p-40, and supplemented with 50 mM NaF, 20 µg/mL aprotinin, 1 mM phenylmethylsulfonyl fluoride, and 2 µg/mL leupeptin). Western blotting analysis were performed as described^54^. Antibody specific for E2A (sc-133074) was purchased from Santa Cruz Biotechnology (Burlingame CA, USA). Antibodies specific for p63 (CY5659), p21 (CY5543), and GAPDH (AB0037) were purchased from Abways (Shanghai, China). Antibodies specific for CyclinE1 (3327) and CyclinD1 (2261) were purchased from Epitomics (Burlingame CA, USA). GAPDH was used as loading control.

### mRNA isolation and quantitative RT-PCR

Total mRNA was extracted using RNA isolation Kit (macherey-nagel) (Düren, Germany) according to the manufacturer’s instructions, and 1 μg RNA template was reverse-transcribed into cDNA by RT-PCR Quick master mix (TOYOBO, Osaka, Japan). cDNA was amplified using SYBR Green Master Mix (Bio-Rad, Hercules CA, USA) by using the primers: E47 Q-PCR primer: Forward: 5′-GAGGAGAAAGACCTGAGGGACC-3′ and Reverse: 5′-ACCTGACACCTTTTCCTCTTCTC-3′; △Np63 Q-PCR primer: Forward: 5′-GAAAACAATGCCCAGACTCAA-3′ and Reverse: 5′-TGCGCGTGGTCTGTGTTA-3′; CCNE1 Q-PCR primer: Forward 5′- TGTGTCCTGGATGTTGACTGCC-3′ and Reverse: 5′- CTCTATGTCGCACCACTGATACC-3′; CCND1 Q-PCR primer: Forward 5′- TCTACACCGACAACTCCATCCG-3′ and Reverse: 5′- TCTGGCATTTTGGAGAGGAAGTG-3′; CDKNIA Q-PCR primer: Forward 5′- AGGTGGACCTGGAGACTCTCAG-3′ and Reverse: 5′- TCCTCTTGGAGAAGATCAGCCG-3′; GAPDH Q-PCR primer: Forward 5′-GGGGAGCCAAAAAGGGTCATCATCT-3′ and Reverse: 5′-GAGGGGCCATCCACAGTCTTCT-3′. Data are expressed as ΔΔCt (fold change).

### MTS assay and colony formation assay

The MTS assay and colony formation assay were used to measure the ability of cell proliferation. For MTS assay, briefly, 1.5 × 10^3^ cells/well (H292 or A549) or 1 × 10^3^ cells/well (FaDu, KYSE150 or H1299) were seeded into in 96-well plates and incubated overnight. 10 μL of MTS (G5421, Promega Corporation, Madison, WI, USA) were added to each well, and then cells were incubated for 1.5 h at 37 °C. The absorption was detected at a wavelength of 490 nm using Varioskan Flash (Thermo, Carlsbad CA, USA).

For colony formation assay, cells were seeded into 6-well plates with a density of 500 per well, with medium changed every 3 days. After culture for 7–9 days, the colonies were fixed with methyl alcohol and stained with 0.1% crystal violet for 20 min, and the number of colonies was counted under the microscope (Nikon, Japan).

### Luciferase reporter assay

Luciferase reporter assays were performed with Secrete-Pair Dual Luminescence Assay Kit (GeneCopoeia, USA) according to the manufacturer’s instructions. Briefly, HEK293T cells were co-transfected with 100 ng of ΔNp63-Gluc-SEAP reporter and an indicated dose of E47 expressing plasmid for 36 h. And then cell culture medium were collected and ΔNp63-Gluc and SEAP activities were measured. The ∆Np63-Gluc activity was normalized to SEAP activity.

### BrdU and PI staining assay

For BrdU staining, briefly, cells were labeled with 10 μM BrdU (B9285, Sigma-Aldrich, St. Louis, USA) for 1 h. Cells were collected and fixed in fixation buffer (4% formaldehyde with 0.1% Triton X-100 in 1 × PBS) for 15 min at room temperature. Cells were then washed twice in flow cytometry staining buffer before the treatment of DNase I at 37 °C for 1 h. Cells were then incubated with 5 μL FITC-conjugated anti-BrdU (364103, Biolegend, San Diego, CA, USA) or isotype control (400137, Biolegend, San Diego, CA, USA) antibody for 30 min at room temperature protected from light. The samples were then analyzed using flow cytometer (Beckman Coulter). For PI staining, cells were collected and fixed in 70% ethanol at −20 °C before the treatment with RNase A and propidium iodide (PI) for 30 min. The cell cycle analyses were performed by using Flow Cytometer (Beckman Coulter).

### ChIP assay

ChIP assays were performed in H292 or A549 cells with ChIP-IT Kit (Active Motif, Carlsbad, CA, USA) using antibodies specific for E2A (sc-133074, Santa Cruz, Burlingame CA, USA) or normal mouse IgG (Invitrogen, Carlsbad CA, USA), according to the manufacturer’s instructions. The primers for the ΔNp63 promoter region using primers were as follows: P1 (nucleotides −2303 to −2167): Forward: 5′-ATTTCACAAATGAGGAATCTGAATCC-3′ and Reverse: 5′-TATCCACTATGGCTACTTGAGAACTT-3′; P2 (nucleotides −1730 to −1572): Forward: 5′-TGGATGATTTTCAGGATCATCCAAGT-3′ and Reverse: 5′- ACCTACTTACAAAATACAGTATAATT-3′; P3 (nucleotides −885 to −734): Forward: 5′-AAGCCAATTGATATCTTATGCTTTA-3′ and Reverse: 5′-CTATTTGAATATTTCTTTTCTCTAT-3′; hSHP (nucleotides −474 to −120): Forward: 5′- CCCCTGGCAGGAATG-3′ and Reverse: 5′- AGGTTAGGCAAACAAGC-3′; negative control (nucleotides −40 to +128): Forward: 5′-TGCCTATAGTTGGGTATATATTA-3′ and Reverse: 5′-AAGAAAGGACAGCAGCATTGAT-3′. The *C*_t_ value of each ChIP sample was normalized to its corresponding input.

### In vivo tumor formation assay

The sample size for animal studies was designed according to previous report^55^. 6-week-old female BALB/C nude mice were purchased (DOSSY EXPERIMENTAL ANIMALS CO.,LTD., Chengdu, China), randomly divided into four groups (*n* = 4/group) and housed under standard conditions. 1 × 10^6^ H292 stable cells in a 100 μL PBS were subcutaneously injected into right flanks (four mice per group). Mice were monitored daily. Tumors were measured by a caliper every 2–3 days and were calculated as Volume = (Length × Width^2^)/2. No blinding test was used in assessing the outcome. When applicable, tumors were dissected, weighed, photographed, and fixed with 4% paraformaldehyde and embedded with paraffin for immunostaining analysis. Tumor volumes and weights were presented as means ± SEM. All animal care and animal experiments in this study were performed in accordance with the institutional ethical guidelines and were approved by the institutional review board of Sichuan University.

### Immunohistological chemistry (IHC) and tissue microarray

Tissue microarray slides of human squamous cell carcinoma specimens (HLug-S030PG02) were purchased (OUTDO, Shanghai, China). Antibodies specific against E2A (sc-133074, Santa Cruz, Burlingame CA, USA) and p63 (CY5659, Abways, Shanghai, China), Ki67 (9449, CST, USA) and cleaved caspase-3 (CC3) (9661, CST, USA) were used for immunohistochemistry (IHC) staining. Immunohistochemical assay was performed as described^25^. Slides were scanned by NanoZoomer (Hamamatsu, Japan). E2A and p63 images were analyzed by calculating the integrated optical density (IOD) using Image-Pro Plus 6.0 (Media Cybernetics, MA, USA), and the average optical density (AOD) was calculated using the formula: AOD = IOD/Area as described^56^. Ki67 and CC3 images were analyzed by calculating the percentage of Ki67/CC3 positive tumor cell^57^.

### Clinical relevance analysis

The mRNA levels of E2A in normal tissues or adenocarcinoma were analyzed using Oncomine dataset “Hao_Esophagus” or “Bhattacharjee_Lung”. The mRNA levels of E2A in normal tissues or squamous cell carcinoma were analyzed using Oncomine dataset “Su_Esophagus” or “TCGA”. Pearson correlation of E47 (E2A) and p63 expression was analyzed using Oncomine dataset “Su_Esophagus” or “Bild_Lung”. Kaplan–Meier plots of overall survival (OS) of human squamous cell carcinoma patients stratified by the E2A mRNA expression levels were analyzed using the Kaplan–Meier survival datasets.

### Statistical analysis

Data from cell culture were performed in three independent experiments. Data were presented as means ± SD. The differences between two groups were performed using the two-tailed unpaired Student’s *t* test. The clinical data were compared after homogeneity tests. *p* < 0.05 was considered statistically significant.

## Supplementary information

Supplemental Figure legends

Supplemental Figure 1

Supplemental Figure 2

## Data Availability

Data and resource are available from the corresponding authors.
