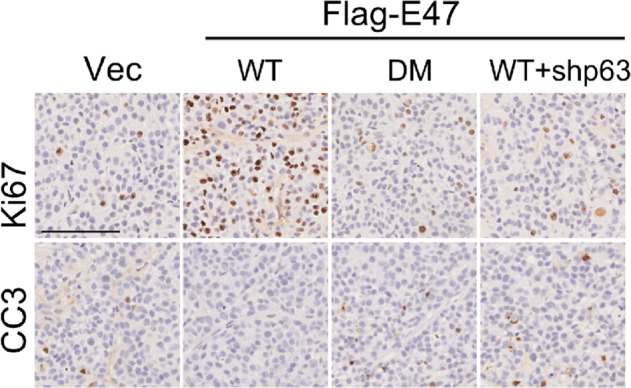# Correction: E47 upregulates ΔNp63α to promote growth of squamous cell carcinoma

**DOI:** 10.1038/s41419-022-05221-w

**Published:** 2022-09-06

**Authors:** Jing Xu, Fengtian Li, Ya Gao, Rongtian Guo, Liangping Ding, Mengyuan Fu, Yong Yi, Hu Chen, Zhi-Xiong Jim Xiao, Mengmeng Niu

**Affiliations:** Center of Growth, Metabolism and Aging, Key Laboratory of Bio-Resource and Eco-Environment, Ministry of Education, College of Life Sciences, Sichuan University, Chengdu, China

**Keywords:** Cell growth, Transcription

Correction to: *Cell Death and Disease* 10.1038/s41419-021-03662-3, published online 08 April 2021

The original version of this article unfortunately contained an error. For Figure 2I, the authors accidentally misplaced the image derived from Immunohistochemistry assays of anti-CC3 in Vec group during the last typesetting process (CDDIS-20-4660R), as inadvertent duplication the image of the WT+ shp63 group. The correct figure can be found below. The authors apologize for the error. The original article has been corrected.