# Supplementary material for: E47 upregulates ΔNp63α to promote growth of squamous cell carcinoma
Source: Cell Death Dis. 2021 Apr 8;12(4):381. doi: 10.1038/s41419-021-03662-3 (PMC8032790; doi:10.1038/s41419-021-03662-3)
Supplement: Supplementary file 1 — Supplemental Figure legends [file 41419_2021_3662_MOESM1_ESM.pdf]

Supplemental Figure legends (S1-S2)

Fig. S1. E47 upregulates ΔNp63α expression and promotes squamous carcinoma cell proliferation.

(A) A549, FaDu or H292 cells were subjected to Western blot analyses.

(B-C) KYSE150 cells stably expressing wild-type Flag-E47, Flag-$E47^{DM}$ mutant ($E47^{A592N/I596D}$) or a vector control were subjected to Colony formation analyses (B) or Western blot analyses (C). Scale bar = 1 cm. Three independent experiments were performed. Data were presented as mean ± SD. **$p < 0.01$.

(D-E) KYSE150 cells stably expressing shRNAs against E2A (+) or GFP (-) were subjected to Western blot analyses (D) or Colony formation analyses (E). Scale bar = 1 cm. Three independent experiments were performed. Data were presented as mean ± SD. **$p < 0.01$.

Fig. S2. Ectopic expression of E47 leads to increased BrdU+ cells in SCC H292 cells, while silencing of E47 leads to increased BrdU+ cells in adenocarcinoma H1299 cells.

(A) H292 stable cells were subjected to BrdU staining and FACS analyses.

(B) H1299 cells stably expressing shRNAs against E2A or GFP were subjected to BrdU staining and FACS analyses. Three independent experiments were performed. Data were presented as mean ± SD. ***$p < 0.001$.
