# Supplementary figures and images for: E47 upregulates ΔNp63α to promote growth of squamous cell carcinoma
Source: Cell Death Dis. 2021 Apr 8;12(4):381. doi: 10.1038/s41419-021-03662-3 (PMC8032790; doi:10.1038/s41419-021-03662-3)

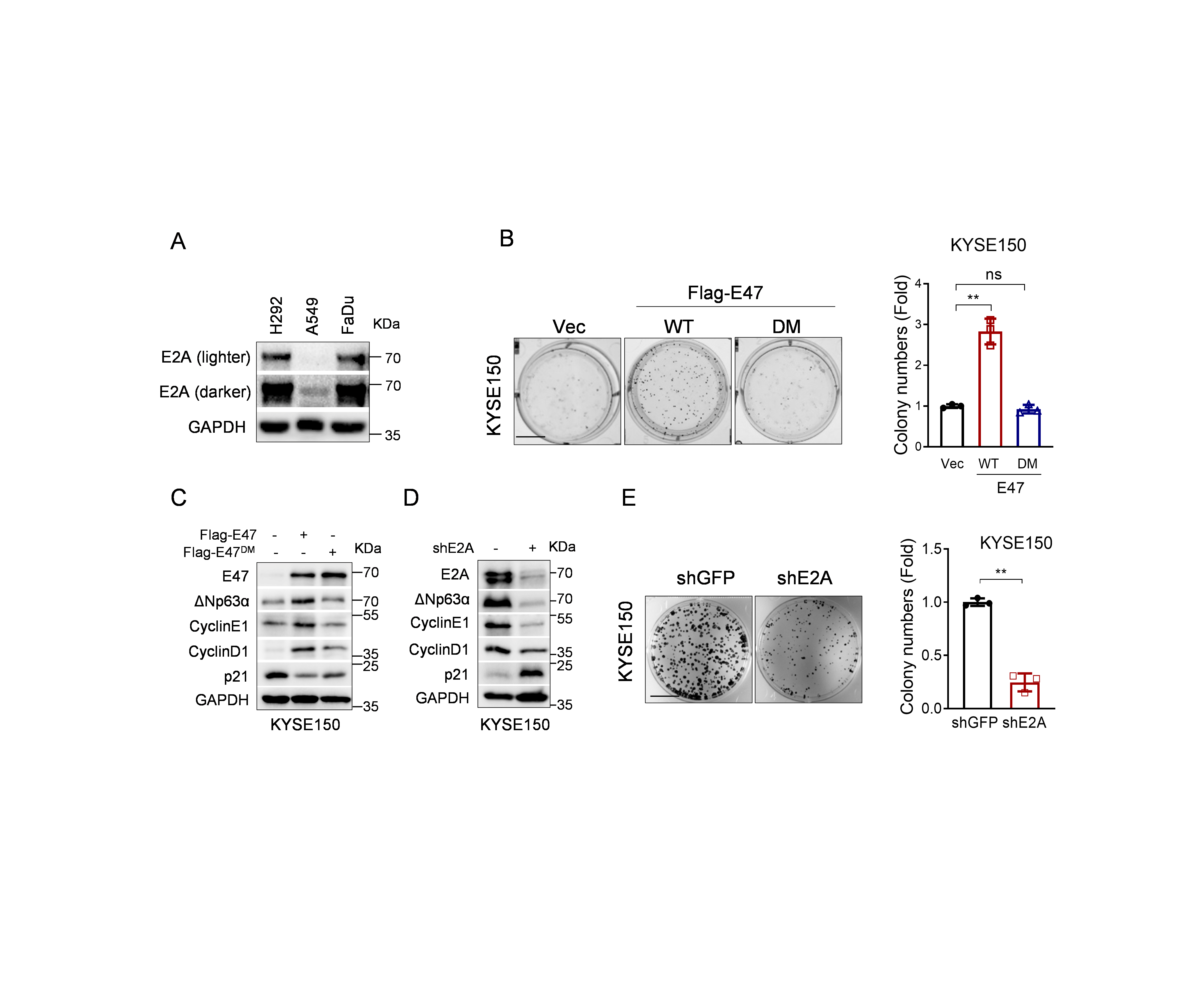

Supplement: Supplementary file 2 — Supplemental Figure 1 [file 41419_2021_3662_MOESM2_ESM.tif]

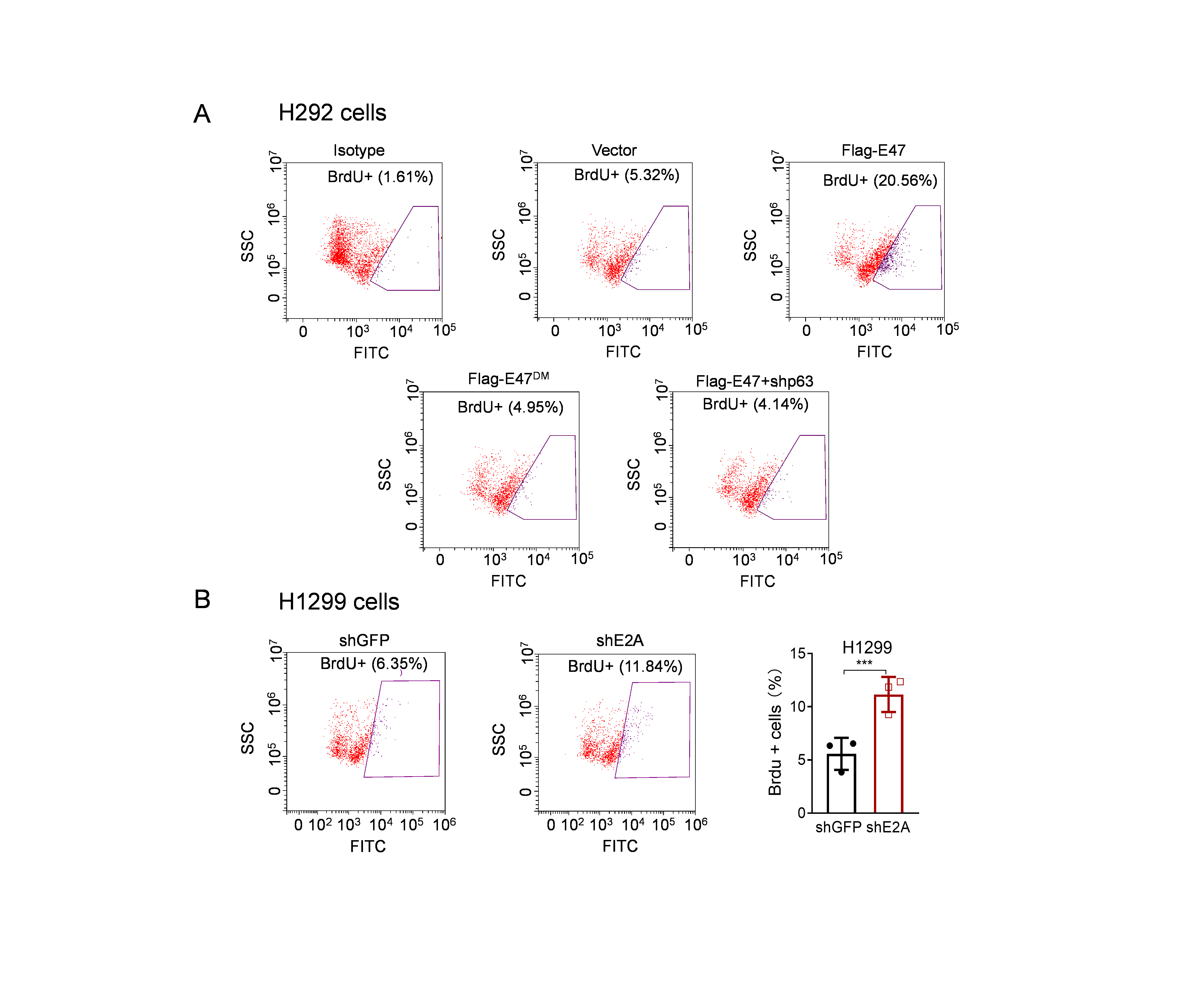

Supplement: Supplementary file 3 — Supplemental Figure 2 [file 41419_2021_3662_MOESM3_ESM.tif]
